# Cultural safety strategies for rural Indigenous palliative care: a scoping review

**DOI:** 10.1186/s12904-019-0404-y

**Published:** 2019-02-14

**Authors:** Kaela Schill, Susana Caxaj

**Affiliations:** 1 Cumming School of Medicine, Community Health Sciences, University of Calgary, Calgary, Alberta Canada; 2 School of Nursing, Faculty of Health and Social Development, Okanagan Campus, University of British Columbia, Kelowna, British Columbia Canada

**Keywords:** Indigenous, Palliative care, Cultural safety, Cultural competency, End of life

## Abstract

**Background:**

There is little scholarship on culturally safe approaches to palliative care, especially for rural Indigenous clients. Thus, it is important to articulate how cultural safety can be enacted to support rural Indigenous Peoples and communities at end of life. We sought to identify strategies described in existing literature that have potential to deepen our understanding of culturally safe approaches to palliative care within rural and small-town settings in Canada.

**Methods:**

We searched for peer-reviewed and grey literature about Indigenous palliative care in rural and small-town settings in Canada, United States, New Zealand, and Australia. Medline, CINAHL, and Embase were searched. We thematically analyzed 22 resulting articles to address our interest in culturally safe approaches to palliative care in rural/small-town and on-reserve contexts.

**Results:**

The following themes were extracted from the literature: symbolic or small gestures; anticipating barriers to care; defer to client, family and community; shared decision-Making; active patient and family involvement; respectful, clear, and culturally appropriate communication; community ownership of services; empower cultural identity, knowledge, and traditions; and, policy.

**Discussion:**

*Culturally competent* practices can improve Indigenous palliative care services; however, they do not result in decolonized care. Strategies include: symbolic or small gestures; anticipating barriers to access; deferring to the client, family, and community members; and, collective decision making and family involvement. *Culturally safe* approaches contribute to institutional or organizational change and decolonized care*.* Strategies include: involvement of patient and family in service planning; reflection about individual and systemic racism; community ownership of services and; recognizing distinct Worldviews that shape care.

**Conclusions:**

*Culturally safe* strategies invite decolonization of care through awareness of colonialism, racism, and discrimination. They invite commitment to building partnerships, power sharing, and decision-making in the delivery of care. C*ulturally competent* activities may catalyze the adoption of a cultural safety framework; however, mislabeling of *cultural competency* as *cultural safety* may contribute to organizational inaction and a watering down of the spirit of cultural safety.

## Background

Calls for improved, culturally appropriate care have emphasized the need for clinicians to consider the background, assumptions, and values of themselves and their patients to provide conscientious, holistic, and relational care [1]. By emphasizing context and difference through a bi-cultural lens, a *culturally safe* approach to care has unique potential to orient clinicians to champion equity, respect and social justice in their practice [2]. Developed by a Maori nurse, Irihapeti Ramsden, as a means of addressing the colonial context shaping the health and wellness of Indigenous peoples in New Zealand [1], it has been adopted by various Indigenous organizations and health care institutions navigating similar social, economic and political realities in North America [3].

As a concept, cultural safety encompasses self-awareness of the clinician’s own culture and an analysis of positional power, including colonial contexts, that can serve to police or restrict cultural norms or values of certain groups [1]. A related but distinct term is cultural competence. Cultural competence is focused on a clinician’s awareness, knowledge, skill and interaction with clients of different cultural groups than their own [4]. While both concepts emphasize the clinician’s self-awareness, a cultural competence lens suggests that cultural groups may have certain fixed or knowable characteristics, and does not involve an interrogation of the larger context in which care is provided or the clinician’s own biases or assumptions [5, 6]. In Canada, cultural safety is a guiding principle for practice by various health professional associations, health authorities, and academic institutions [3, 7, 8]. Yet in the availability of Canadian healthcare services and delivery of care options, there continues to be a paucity of culturally safe approaches for Indigenous clients [9]. This is very much the case in the delivery of palliative care, particularly in rural regions where programs are still in their early stages of development [10]. A literature review by Shahid et al. [11] found that clinician’s limited knowledge of diverse cultural views of death and essentialized approaches to palliative care posed a significant barrier to culturally safe palliative care services and supports. Many Indigenous clients and families in turn, report unmet spiritual and emotional needs, despite it being a priority area of care [12, 13]. This group may be particularly distrusting of advanced care planning which can put them at a disadvantage in navigating treatment options [14]. These occurrences are shaped by a history of colonial injustices of which many ageing Indigenous people are survivors [15, 16] and speaks to the need for culturally safe palliative care.

For rural Indigenous communities access to, and experiences of, palliative care may be complicated by relocation to urban centres, navigating an unfamiliar health care system, and managing with less resources, clinicians, or access to specialty care [17]. Adequate representation of Indigenous community members in clinical roles is often an issue in rural settings which can lead to barriers in cultural understanding, communication, and self-determination for Indigenous families navigating the healthcare system [16]. Thus, it is of utmost importance to articulate how cultural safety can be enacted in a rural palliative care context in order to develop relevant approaches to support these communities at end of life.

We carried out a scoping review and subsequent thematic analysis to identify pathways for culturally safe approaches to palliative care in rural Indigenous contexts. Our guiding question was: *What does a culturally safe palliative approach to Indigenous patient care look like?* This project followed from a prior scoping review in which we analyzed a larger set of literature to determine priorities and challenges for Indigenous-centred palliative care. These results have been previously published [17] . In this paper, we move beyond a simple description of the articles we found through this process, towards a more engaged interpretation of prior literature, developed through a qualitative analytic approach [18]. A key theme emerging from our interpretation suggests that palliative care scholarship, parallel to other areas of healthcare, continues to focus on superficial, rigid, and individualist (rather than structural) conceptualizations of culture which at best encompass a *culturally competent* approach to care. To move towards a *culturally safe* approach to palliative care, strategies to address the continued influence of colonialism and discrimination are needed [19]. This shift will require efforts at the interpersonal, institutional, and policy level, and approaches that promote Indigenous self-determination.

### Historical context of indigenous peoples in Canada

Prior to first contact, the land known today as Canada was inhabited by Indigenous peoples. The social structures, diets, lifestyle, and medical systems developed by Indigenous Peoples enabled them to support health holistically and experience a high quality of life [20, 21]. Landing in a huge and geographically diverse land, the initial settlers who arrived in Canada relied on the support and guidance they received from the First Nations and Inuit for prosperity (e.g., hunting, trapping for profit) and survival [20].

However, first contact brought with it a steep decline in the health of Canada’s First Peoples as they experienced biological and social challenges to their wellbeing. Indigenous peoples were exposed for the first time to European epidemics. Transmitted via trade, dieases such as influenza, typhus, and (perhaps most significantly) smallpox, decimated communities. In some cases, these diseases were spread intentionally via the distribution of infected blankets as a means of germ-warfare resulting in a significant population collapse [22].

Eventually, reserve lands were negotiated via a series of treaties between the Crown and some Indigenous leaders. Through the signing of the treaties, Indigenous leaders agreed to share the land with European settlers in exchange for certain guarantees including (but not limited to) hunting and fishing rights on treaty land, healthcare, education, and cash payments. However, in the early nineteenth century, governmental policy regarding Indigenous peoples became increasingly paternalistic. For example, via the controversial Indian Act of 1876, the federal government took responsibility for the administration of First Nations Status through registration in the Indian Register, thus attempting to legislate First Nations identity, and for the management of First Nations lands, held in trust by the government. With forced assimilation being one of the express goals of colonial policy, the Indian Act enacted a system of Indian Reservations and replaced local government with Band councils, undermining the existing governance structures within the communities and making them increasingly vulnerable to colonial control. This act also made illegal the spiritual practice of ceremonies such as the Potlatch and Sundance in an attempt to further erode First Nations’ identity and enforce assimilation into the settler culture [20].

One of the most horrific methods the federal government employed to achieve the forced assimilation of Indigenous peoples was, as per the Indian Act, mandatory attendance of First Nations and Inuit children at federally funded (and often church-run) Indian Residential Schools. By forcibly removing First Nations children from their families and punishing the children for speaking their language or practicing their traditional teachings, Indian Residential Schools aimed to sever the connection between the children and their culture to “*kill the Indian in the child*” [23]. In recent years, the neglect and sexual, physical, emotional, and spiritual abuse of children at Indian Residential Schools has been well documented [23]. Notably, the last Indian Residential School in Canada closed only 22 years prior to this publication, in 1996. Similarly, the government implemented “*Indian hospitals*” (1920–1960), where Indigenous patients were held and treated separately from the rest of the population [24]. Similar to residential schools, both medical abuse and other forms of abuse have been brought to light in recent years in these institutions, and was part of a “broader colonial project of racial exclusion and segregation”. In addition to the role colonization plays as a social, economic, and political determinants of Indigenous peoples’ health and wellness [25], colonial history is central to understanding Indigenous communities’ experiences with end-of-life because the loss of cultural protocols and practices may complicate grief and pose a barrier to the healing process [26].

### Indigenous health disparities & the social determinants of health

This colonial history has shaped the disparity in health between Indigenous and non-Indigenous peoples in Canada today. For example, Indigenous people are less likely to have a regular medical doctor or to perceive their overall or mental health to be very good or excellent in comparison to non-Indigenous counterparts. Indigenous people also experience a disproportionate burden of chronic disease, infectious diseases, and in the province of BC, notably higher rates of infant mortality [27].

Prior reports indicate many Indigenous communities in Canada do not have access to the same level of health care as other residents in Canada. For example, a report by the Auditor General of Canada in 2015 [28] observed the federal government had failed to ensure adequate training of clinicians, standardization of scope of practice, and systematic documentation and consultation with local communities in their programming. This report concluded initiatives to “resolve interjurisdictional challenges have generally not been effective” (p 23). These jurisdictional complications have a direct impact on the care of Indigenous persons navigating complex chronic illnesses and palliative care services [17].

Contemporary health and social disparities must be understood as reflections of the ongoing damage of colonialism, and despite advocacy by Indigenous communities and leadership Indigenous peoples continue to experience inequities in many social determinants of health and wellness [25]. For example, research indicates many survivors of Indian residential schools experience a lifetime negative impact on their health status that is often compounded by inequitable access to education and lower paying jobs, and thus, increased poverty [29]. Further, inequitable funding and resource allocation to on-reserve communities has resulted in inadequate housing and sanitation, lower water quality standards, and risk of waterborne diseases,, increasing prevalence of adverse health outcomes in some communities [27]. Yet despite growing awareness of the inequity experienced by Indigenous peoples, the latest census data suggests there has been very little change in disparities in income, education and employment experienced by Indigenous populations in Canada [30]. These consistent disparities may be explained by a lack of political will and inaction on prior commitments to improving equity. For instance, the Kelowna Accord of 2005, which set targets to double the number of practicing Indigenous health care professionals and address several health disparities, was not honored or renewed by subsequent federal leaders, resulting in a lack of infrastructure to carry out these changes [27].

Given the significant impact of colonial history on Indigenous health, Wein and Reading [25] championed a shift away from Aboriginal identity as a social determinant of health, and to consider instead the role of colonial history in shaping Indigenous health. They developed three categories of determinants of health and wellness: the Proximal (e.g., health behaviours, employment, income), Intermediate (e.g., health care and educational systems, community resources and capacities, cultural continuity), and Distal (e.g., colonialism, racism, and self-determination) determinants of Aboriginal health. In short, root causes such as colonization, racism, social exclusion, and threats to self-determination all negatively influence the intermediate and proximal determinants of health, ultimately posing a barrier to the health and wellness of Indigenous peoples in Canada today. Cultural safety has been proposed as an approach that addresses the distal determinants of health and wellness, and will be explored in further detail in the following section.

### A transition towards cultural safety

Dialogue around cultural safety has a long history in Canada reflecting changing views on cultural difference, onus for accommodation, and the role of power and privilege in healthcare service planning, delivery, and evaluation [31].

#### Colour-blindness

Prime Minister Pierre Elliot Trudeau’s 1960’s policy of multiculturalism and universalism called for provision of equal services to all people in Canada despite cultural differences. Specifically, the Trudeau government’s 1969 “White Paper” recommended services be “blind” to cultural differences between Indigenous and non-Indigenous people. The aim of this model was to reduce racism and discrimination by treating all people equally. However, the White Paper failed to acknowledge Canada’s colonial history, consequently erasing the impact of colonization on Indigenous peoples’ health and wellbeing, and willfully dismissing the distinct rights, knowledge and culture of Indigenous peoples [32]. By dismissing the unique historical, political, economic, and social context in which the health and wellness of Indigenous peoples is constructed, a ‘colour-blind’ approach ignores racialized inequities, further perpetuating ongoing imbalances of power and privilege [33], the result of which is increased risk for adverse health and wellness outcomes among Indigenous peoples [25].

#### Cultural competence

The push-back to the White Paper included the emergence of an academic body of literature called transculturalism. Scholars from this camp recommended White healthcare professionals strive for an understanding of Indigenous culture and practice, resulting in an approach to care called “cultural competence” [31]. Cultural competence encompasses various practices designed to improve the accessibility and quality of health care for non-White (ethnic/racial ‘minorities’) populations. This term, developed in the 1980s, first focused on improving relationships between non-English speaking immigrants and health care professionals, but since then, several models have been developed to address the particular needs of various groups, including Indigenous peoples [9]. Yet culturally competent frameworks have been under scrutiny by some scholars who suggest that it promotes a rigid and narrow view of culture that may detract from other social determinants of health [34]. Further, it does not prompt health care professionals to consider their own cultural norms and institutional practices that may limit the services available to non-White populations [15].

#### Cultural safety

Irihapeti Ramsden, a Maori nurse in New Zealand, began developing the theory of cultural safety as part of her doctoral work in the 1990’s [35]. A response to what the community saw as inadequate healthcare, Ramsden’s cultural safety was a unique approach to Indigenous health because it: (a) was developed *by* Indigenous communities, *for* Indigenous communities; and, (b) reoriented the conversation around Indigenous health to focus on self-determination and decolonization. In Canada, cultural safety began appearing in the literature in the early 2000’s [36, 37] when organizations such as the National Aboriginal Health Organization and the Aboriginal Nurses Association of Canada began to advocate for including cultural safety in healthcare provider education and health policy [3, 19]. Today, ‘cultural safety’ is a component of training for practitioners across Canada; for example, the First Nation Health Authority partners with regional health authorities in British Columbia to promote cultural safety with an emphasis on cultural humility, a process of recognizing the importance of humbly learning about another’s lived experience. Cultural safety has been defined as a process of power re-distribution that emphasizes providers’ personal exploration of their own privilege and biases, and also as an outcome that is defined by the recipient of care and/or their family [19]. However, the application of the term “cultural safety” both in the literature and in praxis has been a source of confusion and misinterpretation, and concerns have been raised that it has been incorrectly applied to activities better described as culturally competent, thus retreating from the intended objective of decolonization in healthcare services and relationships between providers and patients [38]. The following section will describe the context of palliative care among rural Indigenous populations, and makes the case for cultural safety in palliative care services.

### Palliative Care in Canada and with rural indigenous populations

In Canada, palliative care services represent a patchwork of siloed services in which policies and programs to improve the consistency and standardization of delivery are still in their infancy. To illustrate, as of 2013 only 4 provinces (Ontario, Quebec, British Columbia and Prince Edward Island) had provincial frameworks for the delivery of palliative care [39], with Alberta publishing their provincial framework in 2014 [40]. Further, in 2011, the Parliamentary Committee on Palliative and Compassionate Care (PCPCC) [41] released a report noting that Canada still “falls far short of quality end-of-life care for all,” (p.7) as evidenced by only 16–30% of those who require palliative care actually receiving it.

The PCPCC [41] also observed discrepancies in palliative care service delivery to Indigenous populations when compared to non-Indigenous populations in Canada. Their recommendations included: (i) the need to strengthen capacity in palliative care by building on existing services; (ii) strengthening home care services to improve palliative care options and to better support Indigenous peoples living with chronic illness; (iii) taking action to curb the relocation Elders experience at end of life that keeps them away from their families and; (iv) developing community-based models that facilitate recognition of the unique cultural values, traditions, and languages of Indigenous peoples. Rural communities were also acknowledged as having unique needs and lifestyles that should be acknowledged in palliative care programming. The committee emphasized the preference for rural residents to stay close to home when approaching end of life and a collectivist spirit that may be a key source of support for palliative clients. Further, the need to build on the strengths and resources available in local communities, such as home care services, long term care homes, and local hospice volunteers, was also acknowledged, and points to the need for an engaged culturally safe palliative approach to care [41].

The first phase of our scoping review of rural Indigenous palliative care similarly identified 3 key priorities including local capacity-building, flexibility and multi-sectoral partnerships, and family connections. The 3 key challenges we found included: (i) *staffing issue*s, particularly in regards to limited ability to recruit/retain Indigenous health care workers, and pressures on these individuals because of their scarcity; (ii) *institutional and cultural barriers*, which include jurisdictional service gaps, lack of knowledge of local or culturally-relevant supports, rigid Western biomedical parameters for palliative care, and a lack of recognition of a history of colonization that increased the vulnerability of Indigenous seniors and; (iii) *interpersonal dynamics* such as mistrust, mismatched expectations, poor communication or lack of a common language or understandings, stereotypes, and assumptions that Indigenous families will ‘take care of their own’ [17]. These challenges are shaped by both the current-day realities of the healthcare system, and, by the political and economic realities of many Indigenous families in rural and urban contexts.

A consistent barrier to access for Indigenous populations stems from tensions between provincial and federal bodies, who often dispute jurisdictional responsibilities in the care and treatment of Indigenous patients and families. This may result in clients ‘falling through the cracks’ in seeking economic compensation or time-sensitive treatment. This was the case with Jordan River Anderson, a five-year-old child who spent years up until his death in a hospital needlessly due to federal and provincial disputes as to who was responsible to fund his home care. As a result, complete implementation of Jordan’s Principle, a practice that will ensure that there is no delay in treatment for Indigenous children due to jurisdictional disputes, is the third call to action by the Truth and Reconciliation Commission of Canada [23]. Very few plans for implementation of this principle exist across the country, and unfortunately it has yet to be applied to Indigenous peoples past the age of majority.

Despite many challenges, over the past few decades, community leaders, scholars and clinicians have been active in developing approaches to palliative care that are more culturally appropriate or relevant to Indigenous populations. Examples include Holly and Prince’s community-based palliative care model that highlights community capacity development, cultural competence and safety, participatory action research, ethics, and partnerships in the development of local programs (2011). Organizations like the Canadian Virtual Hospice Society, Pallium, and the Canadian Hospice Palliative Care Association (CHPCA) have also produced tool-kits, videos and training resources over the past 10 years to better meet the unique needs and priorities of Indigenous communities [39]. The following section describes this project’s objectives, as they relate to cultural safety in palliative care services.

### Review objective

Our key objective in this study was to explore what a culturally safe, palliative approach to care looks like in a rural Indigenous context.

## Methods

Our process first involved a general exploration of the body of literature about palliative care in Indigenous rural contexts in an effort to identify gaps in the literature and to identify key concepts. To carry this out, we conducted a scoping review [42, 43]. Following this initial search, we used a data analysis approach similar to that proposed by Whittemore & Kapfl [18] to develop an interpretative understanding of the relevant literature on culturally safe approaches to palliative care (a subset of the larger data-set from the scoping revew). This approach involved extraction of relevant findings and patterns, first through the development of codes and then moving towards a higher level of abstraction. Both authors participated in the initial coding and theme-building and emerging themes were checked against initial codes to establish analytic credibility [44].

### Inclusion criteria

The following inclusion criteria were used: studies about Indigenous palliative care in Canada, United States, New Zealand and Australia, in rural (including on-reserve) and small-town settings. Studies based exclusively in urban settings were excluded. All articles were then screened for their relevance to culturally-safe or competent approaches to palliative care, although authors did not necessarily reference or refer to their work as cultural safety or cultural competency. Rather, inclusion criteria included keywords or significant discussion on topics/concepts of culture (including historical, emotional, spiritual, familial), cultural safety, cultural sensitivity, cultural approaches to care, addressing cultural diversity, or Indigenous/community-led or informed palliative care services. Articles were excluded if they did not address cultural issues or Indigenous-specific considerations or approaches to care.

### Literature search

The search strategy was developed in collaboration with a University of British Colimbia Okanagan research librarian. Medline, the Cumulative Index to Nursing and Allied Health Literature (CINAHL), and the Excerpta Medica Database (Embase) were searched using variations on *Indigenous populations*, *palliative care*, and a *rural* setting. The search was conducted in April 2016, and was limited to English language studies. There were no date limiters. The Initial search included 271 papers. After duplicates were removed, 142 remained. Ninety-eight articles did not meet inclusion criteria (palliative care; rural or remote Aboriginal communities; communities in Canada, United States of America, Australia, or New Zealand). Forty-four were included for full review; 26 were excluded after a thematic analysis was conducted to determine their relevance to cultural approaches to palliative care, specifically, their alignment with inclusion criteria outlined above. Eighteen were included in the qualitative analysis and the reference lists were hand searched for additional literature. There were no study design limiters; however, the articles that were included in the analysis after abstract screening and full review all used a qualitative or secondary (i.e., literature review) research methodology. Search and screening occurred between April 2016–February 2017.

**Fig. 1:**
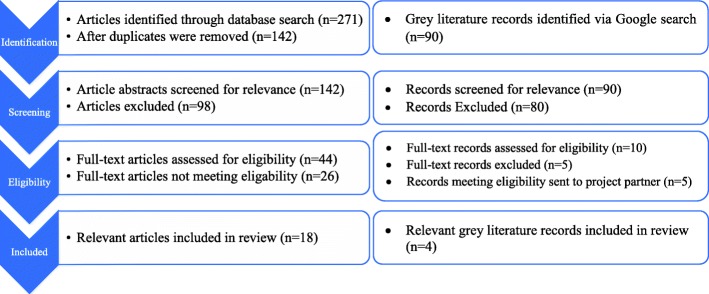
Search Decision Flowchart

For grey literature, Google was searched using the same search terms. The first 10 pages scanned for relevance, and ten pieces were selected for full review. The five most relevant grey literature were sent to the project partners at the First Nation Health Authority for assessment. They recommended four to be included in the final analysis. Analysis was completed by KS and SC. See Fig. 1 for a flow chart of the search decision flow chart.

## Results

### Symbolic or small gestures

Several authors advocate the use of models of care with a basic understanding of Indigenous culture [44, 45] that avoid generalizations or pan-Indigenous approaches [10, 46]. For instance, a basic recognition of unique Indigenous cultural values can be demonstrated through *symbolic or small gestures* that suggest a willingness to accommodate or welcome Indigenous palliative clients. Strategies may include:

- Creating a welcoming space for Indigenous patients through displaying art pieces, or acknowledging and participating in National Aboriginal Day [47];
- Integrating culturally appropriate food in menus [10, 47, 48]; and,
- Providing care that respects spiritual practice in the absence of culturally-specific spiritual care services [49].

### Anticipating barriers to care

Anticipating barriers to access, and in particular those that affect Indigenous peoples inequitably, is important in guiding the provision of Indigenous palliative care. These strategies can include:

- Using a visual analog scale for pain [10, 45, 46];
- Providing a language interpreter or liaison or alternatively, using family members for interpretation when possible [10, 47–50]; and,
- Culturally appropriate informed consent (e.g., oral consent) [51].

### Defer to client, family and community

The literature clearly advocates for clinicians to defer to the client, family and community to ensure a client’s wishes are being honored at end of life. Specifically, the literature prompts clinicians to consider that:

- Patients may need to be dressed in specific clothing during end of life; there may be cultural beliefs around handling of the body (e.g., who is allowed to touch); saying the name or taking photographs of the deceased may be inappropriate [47];
- Some medications may interfere with cultural practices such as ceremony or knowledge transfer [52];
- Dying in a ‘good way’ requires a connection to the land for some Indigenous peoples (e.g., a window visible from the bed or access to outdoors) [10, 47, 48];
- No resuscitation may be requested; lights or sirens may be inappropriate during transport of the body; explicit declaration of imminent death may be inappropriate [49]; and,
- Developing regionally specific, flexible service delivery models, allowing for delivery in home communities to allow for connection to the land, family, spiritual practice, etc. [48, 53, 54].

### Shared decision-making

Strategies that enable shared decision-making with client and family can accommodate the priorities of Indigenous families in palliative care planning. Examples may include:

- Availability of large spaces to accommodate gatherings of large numbers of family or friends [47, 48, 55];
- Giving information to the ‘right’ (i.e., culturally appropriate) person, using family meetings to communicate information, including family or friends identified by the patient or their family [51, 52]; and,
- Developing a conflict resolution process and ongoing involvement after death to avoid blame among family and friends [52, 56].

### Active patient and family involvement

Active involvement of patient and family in service planning, however, goes past shared-decision making to ensure the client, family, and community are considered equal partners with care providers in decision-making*.* The resulting re-orientation of power can promote care that aligns with the cultural values and goals of the client/family/community [45, 46, 49–51, 53, 57, 58]. Examples from the literature include:

- Making time for one on one discussions about care (i.e., “speak less about, and more with”), making time and space for feedback from patients/families to ensure they understand and have the resources and knowledge to actively participate in care [46, 51, 59];
- Respecting decisions not to take medications, respecting and accepting different perspectives on treatment or care [51, 52];
- Educating family on the dying process to facilitate a sense of empowerment [49];
- Adopting culturally appropriate and family-centered communication [55];
- Providing resources to reduce burden of care on family carers (e.g., provision of respite) [49, 57];
- Implementing an Aboriginal health advocate programs to support patient involvement in care and to negotiate power dynamics between patient and provider [50]; and,
- Recruiting and facilitating the participation of healthcare providers, volunteers, or family/friends of the same cultural background [44, 52, 53, 59].

### Respectful, clear, and culturally appropriate communication

At the individual level, the need for providers and administrators to use *respectful, clear, and culturally appropriate communication* has been emphasized [45, 49, 51–53, 57]. For example:

- Incorporating local Elders into the planning and delivery of cultural safety training (e.g., by using a tea room style chat) to enhance the local relevance of the teachings [50];
- To promote healthy communication, cultural safety training ought to facilitate reflection about individual and systemic forms of racism and judgements [49]; and,
- While individual clinicians must be open to change in order to enact principles of cultural safety and develop trust with their clients, relationships of trust and knowledge sharing must also be demonstrated at an institutional level [50, 56, 60, 61].

### Community ownership of services

The literature emphasizes the importance of community ownership of palliative services to ensure the needs of clients, families, and communities are adequately met. To achieve this, provision of services must be driven by the needs of the community [10, 61] and built on community strengths and culture [56]. For example:

- Universities and hospitals may partner with community organizations or Indigenous leadership to provide support and resources for capacity building in program development, delivery, and evaluation [49, 61]
- For communities or organizations with fewer established healthcare programs or resources, a first step may be to engage a community advisory committee or liaison in service planning and delivery [61] combined with the explicit goal of building capacity for increased community ownership over time.

### Empower cultural identity, knowledge, and traditions

Effective palliative care approaches are those that empower cultural identity, knowledge, and traditions [51, 54, 55, 59]. Empowerment means that cultural identity, knowledge, and traditions are valued as legitimate sources of knowledge and expertise during the palliative care process and are essential to decision-making. Strategies from the literature that can enhance the empowerment of cultural identity, knowledge, and traditions in palliative care include:

- Educational programs that explore Indigenous models of care and critically evaluate the Western model of care and its underlying assumptions; and,
- Transcending acceptance of traditional medicine, instead acknowledging the inherent value of an Indigenous approach to care [10, 51, 53, 56].

### Policy

Culturally appropriate and informed health care policy is necessary to enact in Indigenous palliative care, and in many cases is still lacking. Some examples of policy that might improve quality of palliative care for Indigenous clients, their families and communities include:

- Culturally appropriate certification and service protocol, with flexibility to reflect cultural values [49, 57];
- Institutional recognition of the family role in dying and care [46];
- Including Indigenous peoples in governance and decision-making structures [62]; and,
- Prioritizing funding of community based palliative care services [48].

## Discussion

**Fig. 2:**
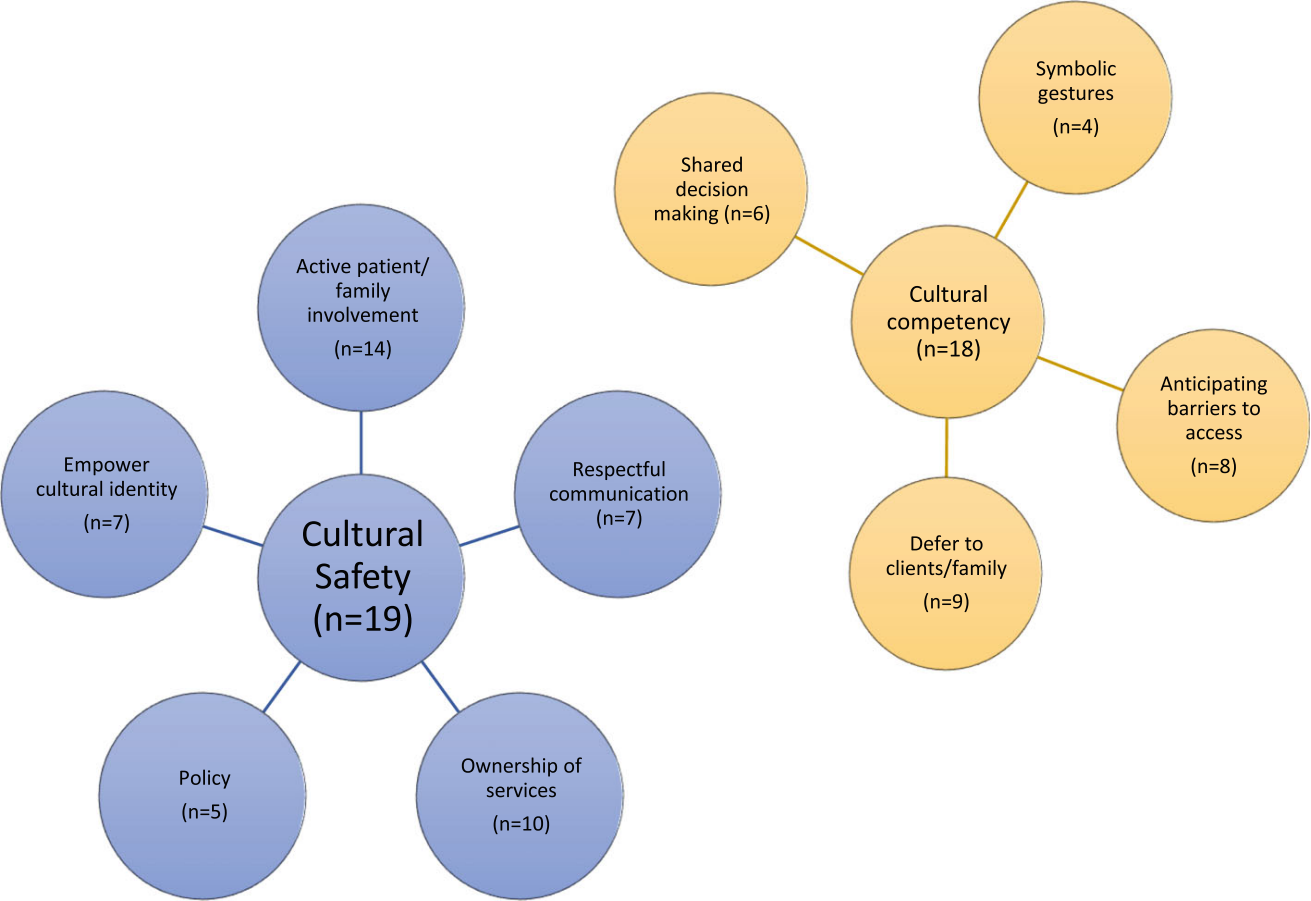
Map of scoping review results

Our analysis of the literature revealed many recommendations to improve palliative care services for Indigenous clients and families. Yet not all these strategies reflect a culturally safe approach. Rather, some may better be described as providing *culturally competent* palliative care. Here, we review these different recommendations and suggest their categorization as either cultural *competence* or cultural *safety*, to build clarity between the terms. In addition, we discuss substantive implications and considerations for enacting these strategies. See Fig. 2 for a map of the results of this scoping review – please note, the result groupings in the map are not mutually exclusive, and one paper may be included in multiple groups and/or sub-groups.

Firstly, we argue that *culturally competent* practices are distinct from *culturally safe* approaches to palliative care. Culturally competent practices in providing palliative care for Indigenous populations may include: (a) symbolic or small gestures; (b) anticipating barriers to access; (c) deferring to the client, family and community members and; (d) facilitating collective decision making and family involvement. While they have the potential to improve palliative care services provided to Indigenous patients, they cannot transform the institutional norms or assumptions that are essential to Indigenous-centred, decolonized care. Nor do culturally competent approaches implore the provider to practice self-reflection to decolonize their practice.

Secondly, we suggest that *culturally safe* approaches are those with the potential to lead to profound institutional or organizational changes and may depend on clinicians having a heightened level of awareness about colonial and political factors shaping care dynamics. Further, a central tenant of culturally safe approaches is the decolonization of the provider’s practice through personal self-reflection about their own privilege, culture, assumptions, or biases that result in culturally unsafe care. Culturally safe strategies include: (a) active involvement of patient and family in service planning; (b) reflection about individual and systemic forms of racism and judgements; (c) community ownership of services and; (d) recognizing distinct Worldviews that shape palliative care.

### Cultural competence: Recognizing difference & accommodation

A cultural competency approach educates non-Indigenous healthcare workers about the cultural practices and worldviews of Indigenous peoples. Once the clinician or health care institution is aware that Indigenous peoples may hold unique cultural values or have unique needs or practices related to their health and wellbeing, they are able to enact this awareness in their day-to- day care through the incorporation of *small or symbolic gestures*. A strength of these strategies is that they can catalyze awareness and acknowledge cultural difference, perhaps normalizing differences in so far as clinicians are expected to accommodate some Indigenous peoples’ preferences. On the other hand, these strategies do not, in and of themselves, create opportunities for clinicians to gain insight into their own cultural values or beliefs, nor do they orient clinicians and administrators to consider the impact of colonialism or discrimination in shaping care.

It is possible that administrators alone hold some understanding of the implications of *small or symbolic gestures* (as they pertain to catalyzing awareness and normalizing cultural differences) and clinicians lack this awareness and are simply complying with institutional requirements of practice. If culturally competent strategies are implemented using top-down standards of care, they may pose a risk to Indigenous clients if clinicians are reluctant to enact them, or, are unaware of the meaning behind such accommodations. Further, without understanding cultural differences within a context of forced assimilation and a history of colonialism, clinicians may see Indigenous clients’ differences as a hindrance to routine care, rather than an opportunity to rethink their assumptions about what constitutes quality care. Nonetheless, integrating an awareness of cultural difference in day-to-day practice using principles of cultural competence, has potential to improve the fit of standard clinical assessments and clinical experiences for Indigenous clients and their families [4].

*Anticipating barriers of care* that are specific to Indigenous Peoples is an important part of increasing equitable access to palliative care services. However, such culturally competent strategies may inadvertently catalyze stigma or paternalistic views about Indigenous peoples if they take a deficit-based approach, focusing solely on inequity without understanding the initial mechanisms (historic, economic, social, or political) that underpin the inequity. Further, many scholars suggest that cultural competency essentializes racialized minorities, erasing the diversity and complexity within groups [63]. Therefore, we suggest that while anticipating barriers in care should be applied in Indigenous palliative care planning and implementation, health care institutions should be aware that without a profound orientation as to the root-causes shaping these disadvantages in care, an Indigenous-centered approach to care may be limited. Further, it is imperative to note that culture does not equate to ethnicity; rather, there are many factors such as language, traditional knowledge, or cultural practice that can contribute to an individual’s culture.

One strategy employed in culturally appropriate care is *deferring to the client, family and community* by centring the preferences, beliefs and values of families in care planning and provision. A culturally competent framework informs clinicians about potential practices or preferences they may not be familiar with, and readies the provider to support the client/family/community by enacting them. A critique of these types of recommendations for culturally competent care is that they may be too prescriptive and can exacerbate a clinician’s tendency to exoticize Indigenous peoples as the ‘other,’ instead of engaging relationally with families to consider their unique preferences [6]. With this mindset, clinicians may disengage from developing culturally responsive care. For instance, prior research indicates many hospice care centres are not motivated to adopt their services to attract Indigenous or racialized clients, assuming these groups prefer to provide care amongst themselves [13, 14]. However, a non-Indigenous clinician may benefit from some basic examples of ways that some Indigenous approach to death and dying may differ from the cultural norms the clinician is accustomed to [51]. We would suggest that as long as clinicians use their general knowledge of cultural practices as a point of entry to express curiosity about a client and family’s wishes, a culturally competent framework may contribute to more Indigenous-centred palliative care provision.

Palliative care is a community and family experience in many Indigenous cultures [52, 55]. As such, the need to understand and respect cultural rules around family involvement in care [45, 50, 53, 56] has been identified as a key principle of culturally competent palliative care. One of the ways this can be enacted in culturally competent approaches is via *shared decision-making*. Notably, we observe shared decision-making strategies, although still focused on cultural differences and accommodation, result in provider/client/family partnerships that provide opportunities to challenge normative institutional practices or clinician assumptions. Perhaps by focusing on building relational accountability between a provider and clients/family/community, power-sharing in care planning can begin to develop. Thus, genuine partnerships in palliative care may be the culturally competent strategy with the most potential to be transformative and set the stage for a culturally safe approach to palliative care.

### Cultural safety

A culturally safe approach reorients the power dynamics between clinicians and the client/family/community, and lead to recognition of the systemic and historical factors shaping palliative care experiences for Indigenous clients. Specifically, cultural safety requires the practitioner to practice ongoing self-reflection about their own culture, beliefs, assumptions, and biases. Culturally safe approaches may consequently bring up feelings of personal discomfort and/or organizational push-back as normative assumptions and practices are challenged [1]. Yet ultimately, we argue that integrating culturally-safe approaches into palliative care delivery can support the self-determination of Indigenous clients, families, and communities. Because culturally safe approaches centre around reorienting the power dynamic between provider and client/family/community, an important feature of culturally safe outcomes is that they are defined by the client/family/community themselves [1]. That is to say, it is not for the practitioner to determine whether their practice is culturally safe; rather, the mechanism of validation is the client/family/community.

Going past a superficial understanding of Indigenous culture, *active involvement of patient and family in service planning* is an essential part of culturally safe care in that it aims to empower Indigenous clients/families/communities to determine the type of palliative care that is more relevant to them. This orientation to care requires clinicians to create space for collective decision-making and facilitating exchange of knowledge that meets the unique needs of Indigenous families and communities. Prior writes, “the “culturally appropriate” mantra of [care] misses the point unless there is a deeper engagement of Aboriginal people in their own health care, so that real choices are possible and the significance of culture is understood and respected” [55]. Thus, it is essential not to view these activities as inherently culturally safe, but rather the real potential of these approaches depends on developing opportunities for reciprocal exchange of knowledge and power in the development of a palliative approach to care. Recognizing that it is the client or community alone that can determine if cultural safety is occurring [62] is essential in culturally safe approaches. At the individual level, the need for providers and administrators to use *respectful, clear, and culturally appropriate communication* has been emphasized as important in Indigenous palliative care [45, 49, 51–53, 57]. Respectful, clear, and culturally appropriate communication is necessary for cultural safety by ensuring the client and their family/community is empowered to be actively involved in service planning.

Culturally safe approaches to care are those that *empower cultural identity, knowledge, and traditions* [51, 54, 55, 59]. Western models of care should not be imposed on Indigenous clients; as Prior [55] writes, “A culture-centred approach… is not to divert Aboriginal patients away from conventional methods, but rather to negotiate a balance between the different cultural paradigms…” Recognizing distinct Worldviews that shape palliative care services, and the unique strengths Indigenous and non-Indigenous perspective can bring to care can be a powerful strategy to develop cultural safety [55, 59].

The establishment of trust and reciprocal, respectful relationships at the institutional level is also part of the transformative nature of cultural safety. *Community ownership of services* is an important aspect of culturally safe care [51, 59], with self-governance as the ultimate goal. Administration can contribute to community ownership by promoting and actively developing interdisciplinary and multi-sectoral relationships. At both the individual and systemic level, continuity of care and sustained engagement is crucial to maintaining trust and respectful relationships, and community ownership of knowledge must be honored [61].

*Policy* that aims to empower the cultural identity and self-determination of communities and individuals may be essential to delivering culturally-safe and relevant palliative care. In addition, creating pathways for community ownership of programs and services, and establishing protocols to redistribute power between healthcare providers and patients/families will help to achieve cultural safety in healthcare settings.

## Conclusions

Our review of the literature revealed various strategies to improve the relevance and cultural appropriateness of palliative care for Indigenous clients. We outlined two types of recommendations - those that aligned with a culturally competent framework, and those that were more oriented towards cultural safety. Although our search was focused on rural Indigenous populations, because of the lack of specificity to rural contexts of recommended activities these initiatives are likely applicable to other contexts. Yet more research is required in this area.

Culturally competent strategies focused on building opportunities or creating space to accommodate the unique values and traditions of Indigenous patients, families and communities. One strength of these types of approaches is they coach non-Indigenous clinicians to challenge assumptions of universality and consider how cultural difference and historical context may shape care preferences of their Indigenous clients. One key risk of culturally competent approaches is that without an awareness of provider privilege and power, institutional norms, or historical oppression, they may enforce simplistic stereotypes, essentialization, and stigma of Indigenous peoples. Given the diverse spiritual and cultural practice between Indigenous communities, cultural competence will only be a value added if it is applied with a great deal of humility and genuine curiosity for the individuality and uniqueness of each client. Partnered approaches to care may enable clinicians to transition towards a culturally safe approach to palliative care because partnerships may prompt the individual to consider power at the interpersonal level.

Culturally safe strategies in palliative care emphasize the need for individual and institutional awareness of colonialism, racism, and discrimination, requiring providers to practice self-reflection as part of decolonizing their own practice. They invite active commitment to building partnerships that enable clients and clinicians to share power and decision-making in the delivery of care. Particularly relevant to palliative care, a culturally safe approach to care must move beyond mere accommodation of preferences towards embracing the unique value of Indigenous knowledge systems and their contribution to quality, patient-centered care, and must be validated as culturally safe by the recipient(s) of care. Community ownership of services and policies that enable Indigenous self-determination and honor Indigenous value-systems may be most important in establishing culturally safe palliative care. Such strategies require buy-in and support of multiple sectors and levels of government. Processes to achieve buy-in will likely be very politically charged and challenging for some actors to accept in light of Canada’s colonial history.

Seemingly *culturally competent* activities may be enacted in a way that can catalyze or build culturally safe approaches, so their categorization as such should not be regarded as reinforcing a cultural competence/safety binary, or, cultural competence as de facto inferior. Rather, *culturally competent* strategies require complex planning to propel an institutional commitment towards *culturally safety*. In fact, actors may report enacting a culturally safe approach to care despite lacking the institutional commitment, reflexivity, or longevity required to be accurately classified as cultural safety. This misnaming can contribute to organizational inaction and a watering down of the spirit of cultural safety.

Thus, our categorization of such recommendations is not prescribed, rather, it reflects our attention to principles of cultural safety that we feel are essential to centering Indigenous perspectives in the provision of palliative care. Ultimately, both cultural safety and cultural competence in rural palliative care must be assessed by the extent to which they honor each Indigenous individual, family and community’s values and vision for self-determination.

### Limitations

Unfortunately, the unique ways in which rurality and cultural safety intersect in palliative care was not made explicitly clear in the literature. Furthermore, the usage and definitions of cultural safety and related terms were not consistently found in reviewed articles. Future research ought to examine the ways in which cultural safety is applied differently and distinctly in rural and remote settings, compared to urban settings, and in addition, document the different definitions of cultural safety and related terms available in published literature.

## References

[1] Papps E, Ramsden I (1996). Cultural safety in nursing: the New Zealand experience. Int J Qual Heal Care.

[2] Mcgough S, Wynaden D, Wright M. Experience of providing cultural safety in mental health to aboriginal patients: a grounded theory study. Int J Ment Health Nurs. 2017. 10.1111/inm.12310 28165178

[3] Hart-Wasekeesikaw F, Gregory D (2009). Cultural competence and cultural safety in nursing education - a framework of first nations, Inuit and Metis Nursing. Ottawa, ON.

[4] Campinha-Bacote J (2002). The process of cultural competence in the delivery of healthcare services: a model of care. J Transcult Nurs.

[5] Gerlach AJ (2012). A critical reflection on the concept of cultural safety. Can J Occup Ther.

[6] Gustafson DL (2005). Transcultural nursing theory from a critical cultural perspective. Adv Nurs Sci.

[7] First Nations Health Authority (2013). Cultural Safety Definitions.

[8] Provincial Health Services Authority. San’yas Indigenous Cultural Safety Training. Provincial Health Service Authority in British Columbia, Canada. http://www.sanyas.ca. Accessed 1 Feb 2018.

[9] Clifford A, McCalman J, Bainbridge R, Tsey K (2015). Interventions to improve cultural competency in health care for indigenous peoples of Australia, New Zealand, Canada and the USA: a systematic review. Int J Qual Heal Care..

[10] Castleden H, Crooks VA, Hanlon N, Schuurman N (2010). Providers’ perceptions of aboriginal palliative care in British Columbia’s rural interior. Health Soc Care Community.

[11] Shahid S, Taylor EV, Cheetham S, Woods JA, Aoun SM, Thompson SC (2018). Key features of palliative care service delivery to indigenous peoples in Australia, New Zealand, Canada and the United States: a comprehensive review. BMC Palliat Care.

[12] Duggleby W, Kuchera S, MacLeod R, Holyoke P, Scott T, Holtslander L (2015). Indigenous people’s experiences at the end of life. Palliat Support Care.

[13] Shahid S, Bessarab D, van Schaik KD, Aoun SM, Thompson SC (2013). Improving palliative care outcomes for aboriginal Australians: service providers’ perspectives. BMC Palliat Care.

[14] Kelly L, Minty A (2007). End-of-life issues for aboriginal patients: a literature review. Can Fam Physician.

[15] Browne AJ, Smye VL, Varcoe C (2005). The relevance of postcolonial theoretical perspectives to research in aboriginal health. CJNR (Canadian J Nurs Res).

[16] Castleden H, Crooks VA, Sloan Morgan V, Schuurman N, Hanlon N (2009). Inter tribal health Authority. Dialogues on Aboriginal-focused hospice palliative care in rural and remote British Columbia.

[17] Caxaj CS, Schill K, Janke R. Priorities and challenges for a palliative approach to care for rural indigenous populations: a scoping review. Heal Soc Care Community. 2017. 10.1111/hsc.12469 28703394

[18] Whittemore R, Knafl K (2005). The integrative review: updated methodology. J Adv Nurs.

[19] National Aboriginal Health Organization (2006). Fact sheet: cultural safety.

[20] Erasmus G, Dussault R. Report of the Royal Commission on Aboriginal Peoples. Ottawa: The Royal Commission on Aboriginal Peoples; 1996.

[21] First Nations Health Council (2011). Implementing the vision: BC first nations health governance.

[22] Waldman C, Braun M. Atlas of the North American Indian. Ney York: Facts on File; 2009.

[23] TRC. Honouring the Truth , Reconciling for the Future. 2015.

[24] Lux MK (2010). Care for the ‘racially careless’: Indian hospitals in the Canadian west, 1920–1950s. Can Hist Rev.

[25] Wien F, Reading C. Health Inequalities and the Social Determinants of Aboriginal Peoples’ Health. 2009. https://www.ccnsa-nccah.ca/docs/determinants/RPT-HealthInequalities-Reading-Wien-EN.pdf.

[26] Hampton M, Baydala A, Bourassa C, McKay-McNabb K, Placsko C, Goodwill K (2010). Completing the circle: elders speak about end-of-life care with aboriginal families in Canada. J Palliat Care.

[27] Richmond CAM, Cook C (2016). Creating conditions for Canadian aboriginal health equity: the promise of healthy public policy. Public Health Rev.

[28] Office of the Auditor General of Canada. Reports of the Auditor General of Canada: Access to Health Services for Remote First Nations Communities. Ottawa: Office of the Auditor General of Canada; 2015. http://epub.sub.uni-hamburg.de/epub/volltexte/2015/48265/pdf/parl_oag_201504_04_e.pdf.

[29] Kaspar V (2014). The lifetime effect of residential school attendance on indigenous health status. Am J Public Health.

[30] Mitrou F, Cooke M, Lawrence D, Povah D, Mobilia E, Guimond E (2014). Gaps in indigenous disadvantage not closing: a census cohort study of social determinants of health in Australia, Canada, and New Zealand from 1981–2006. BMC Public Health.

[31] Brascoupe S, Waters C (2009). Cultural safety: exploring the applicability of the concept of cultural safety to aboriginal health and community wellness. J Aborig Health.

[32] Fitzmaurice K (2011). Transgressing the Boundaries of Native Studies: Traces of “White Paper” Policy in Academic Patterns of Indigenization. Can J Native Stud.

[33] Gallagher CA (2003). Color-blind privilege: The social and political functions of erasing the color line in post race America. Race, Gend Cl.

[34] Carpenter-Song EA, Schwallie MN, Longhofer J (2007). Cultural competence reexamined: critique and directions for the future. Psychiatr Serv.

[35] Ramsden I (1993). Cultural safety in nursing education in Aotearoa (New Zealand). Nurs Prax N Z.

[36] Smye V, Browne A (2002). Cultural safety’and the analysis of health policy affecting aboriginal people. Nurse Res.

[37] Browne AJ, Fiske J-A, Thomas G (2000). First nations women’s encounters with mainstream health care services & systems.

[38] Browne AJ, Varcoe C, Smye V, Reimer-Kirkham S, Lynam MJ, Wong S (2009). Cultural safety and the challenges of translating critically oriented knowledge in practice. Nurs Philos.

[39] Canadian hospice Palliative Care Association. Palliative Care in the Community: An Environmental Scan of Frameworks and Indicators. 2013;:1–60. file:///C:/Users/Keala/Downloads/TWF-Environmental Scan Report EN FINAL.pdf.

[40] Alberta Health Services (2014). Palliative and End-of-Life Care: Alberta Provincial Framework.

[41] Parliamentary Committee on Palliative and Compassionate Care (2011). Not To Be Forgotten.

[42] Arksey H, O’Malley L (2005). Scoping studies: towards a methodological framework. Int J Soc Res Methodol.

[43] Peters MD, Godfrey CM, Khalil H, Mcinerney P, Parker D, Soares CB. Guidance for conducting systematic scoping reviews. Int J Evid Based Healthc. 2015;13:141–6. 10.1097/XEB.0000000000000050 26134548

[44] Tracy SJ (2010). Qualitative quality: eight “big-tent” criteria for excellent qualitative research. Qual Inq.

[45] Finke B, Bowannie T, Kitzes J (2004). Palliative care in the Pueblo of Zuni. J Palliat Med.

[46] Rix EF, Barclay L, Stirling J, Tong A, Wilson S. ‘Beats the alternative but it messes up your life’: Aboriginal people's experience of haemodialysis in rural Australia. BMJ Open 2014;4. http://bmjopen.bmj.com/content/4/9/e005945.abstract. 10.1136/bmjopen-2014-005945 PMC4166141 25231493

[47] McGrath P, Ogilvie KF, Rayner RD, Holewa HF, Patton MAS (2005). The’right story’to the’right person’: communication issues in end-of-life care for indigenous people. Aust Health Rev.

[48] Anderson K, Cunningham J, Devitt J, Cass A (2013). The IMPAKT study: using qualitative research to explore the impact of end-stage kidney disease and its treatments on aboriginal and Torres Strait islander Australians. Kidney Int Suppl.

[49] Rix EF, Barclay L, Wilson S, Stirling J, Tong A (2013). Service providers’ perspectives, attitudes and beliefs on health services delivery for aboriginal people receiving haemodialysis in rural Australia: a qualitative study. BMJ Open.

[50] Rix EF, Barclay L, Stirling J, Tong A, Wilson S (2015). The perspectives of aboriginal patients and their health care providers on improving the quality of hemodialysis services: a qualitative study. Hemodial Int.

[51] Brooke NJ (2011). Needs of aboriginal and Torres Strait islander clients residing in Australian residential aged-care facilities. Aust J Rural Health.

[52] Kelly L, Linkewich B, Cromarty H, St Pierre-Hansen N, Antone I, Gilles C (2009). Palliative care of first nations people: a qualitative study of bereaved family members. Can Fam Physician.

[53] Frey R, Gott M, Raphael D, Black S, Teleo-Hope L, Lee H (2013). “Where do I go from here”? A cultural perspective on challenges to the use of hospice services. Heal Soc Care Community.

[54] McGrath PD, Patton MAS, Ogilvie KF, Rayner RD (2007). The case for aboriginal health workers in palliative care. Aust Health Rev.

[55] Prior D (2009). The meaning of cancer for Australian Aboriginal women; changing the focus of cancer nursing. Eur J Oncol Nurs.

[56] McGrath P (2006). The biggest worry’: research findings on pain management for aboriginal peoples in Northern Territory, Australia. Rural Remote Health.

[57] Li-Chin Shih RNMN, Michelle Honey RN (2011). The impact of dialysis on rurally based Maori and their whanau/families. Nurs Prax New Zeal.

[58] Smith T (2012). A long way from home: Access to cancer care for rural Australians. Radiography.

[59] Poroch NC (2012). Kurunpa: keeping spirit on country. Heal Sociol Rev.

[60] McGrath P (2007). ‘I don’t want to be in that big city; this is my country here’: research findings on aboriginal peoples’ preference to die at home. Aust J Rural Health.

[61] McGrath P, Holewa H, Kail-Buckley S (2007). “They Should Come Out Here ...”: Research Findings on Lack of Local Palliative Care Services for Australian Aboriginal People. Am J Hosp Palliat Med.

[62] Richardson S, Williams T (2007). Why is cultural safety essential in health care. Med L.

[63] Mohammed SA (2006). Moving beyond the “exotic”: applying postcolonial theory in health research. Adv Nurs Sci.

